# Post-Spinal Anesthesia Intradural Extramedullary Arachnoid Cyst Formation: A Case Report

**DOI:** 10.7759/cureus.43199

**Published:** 2023-08-09

**Authors:** Shumaila S Malik, Hadi Shah, Maheen Nasir, Safdar A Malik, Fahad Malik

**Affiliations:** 1 Radiology, Alnoor Diagnostic Center, Lahore, PAK; 2 Radiology, CMH Lahore Medical College and Institute of Dentistry, Lahore, PAK

**Keywords:** lower back pain (lbp), secondary intradural spinal arachnoid cyst, spinal cord, spinal anaesthesia, intradural extramedullary arachnoid cyst

## Abstract

The formation of an intradural extramedullary arachnoid cyst is a rare complication of spinal anesthesia. We present a case of a 34-year-old female patient who developed neurological symptoms including a bilateral reduction in sensation and strength in the lower limbs following a C-section under spinal anesthesia. MRI of the thoracic spine revealed a lesion at the level of T11/T12 and the upper limit of the L1 vertebral body, which was pushing the cord to the side, and a diagnosis of the intradural extramedullary arachnoid cyst was established. The prognosis for this condition is good and surgical excision leads to resolution of the symptoms. Proper and prompt diagnosis is crucial to rule out other differentials and prevent permanent neurological damage in these patients.

## Introduction

Lower back pain is a common complaint among adults and can result from various underlying conditions [1]. One such condition is intradural extramedullary arachnoid cyst formation, which can develop following spinal anesthesia [2]. Arachnoid cysts have diverse etiologies. It may have autosomal recessive inheritance and may be associated with other congenital anomalies like meningocele, syringomyelia, neurofibromatosis, Marfan syndrome, or corpus callosal agenesis [3,4].

Arachnoid cysts are commonly encountered cystic lesions within the central nervous system, which are increasingly identified through advanced neuroimaging modalities. These lesions are considered to be incidental findings and are thought to arise from congenital malformations of the arachnoid membrane [5]. Arachnoid cysts within the spinal column are detected in approximately 25% of cases, with the majority located within the thoracic region (77%). While they are usually asymptomatic, these cysts may infrequently cause spinal cord compression, particularly in the thoracic region. They are commonly found within the middle to lower thoracic spine but have also been identified in the lumbar, lumbosacral, and thoracolumbar regions [6-8].

Spinal anesthesia is a commonly used technique for a variety of surgical procedures. While generally considered safe, it is not without risks. One rare complication that has been reported in the literature is the formation of intradural extramedullary arachnoid cysts following spinal anesthesia. These cysts can cause a range of symptoms, including lower back pain, radiculopathy, and myelopathy [2]. The pathogenesis of these cysts is not well understood, but it is thought to be related to the disruption of the arachnoid membrane during the spinal anesthesia procedure [9]. Diagnosis is typically made through MRI [10], and treatment may involve surgical intervention [11].

## Case presentation

The patient was a 34-year-old female with no significant medical history before her pregnancy. She had had an uneventful pregnancy and undergone a C-section six months ago under spinal anesthesia for the delivery of her first child. The C-section procedure had been reportedly successful, and there had been no immediate postoperative complications.

The patient presented to the clinic with a complaint of persistent lower back pain for the past six months. She described the pain as a dull, aching sensation in the lower back, which occasionally radiated to both legs. Along with the pain, she reported experiencing numbness in both lower limbs. The patient expressed concern about the gradual worsening of her symptoms over time and the limited relief obtained from over-the-counter pain medications. During the physical examination, the patient's vital signs were within normal limits. Her general appearance was unremarkable, and there were no signs of distress. The patient demonstrated reduced sensations in bilateral lower limbs below the knee level. She denied any urinary or fecal incontinence. She did not report any difficulties in initiating or stopping urine flow.

**Figure 1:**
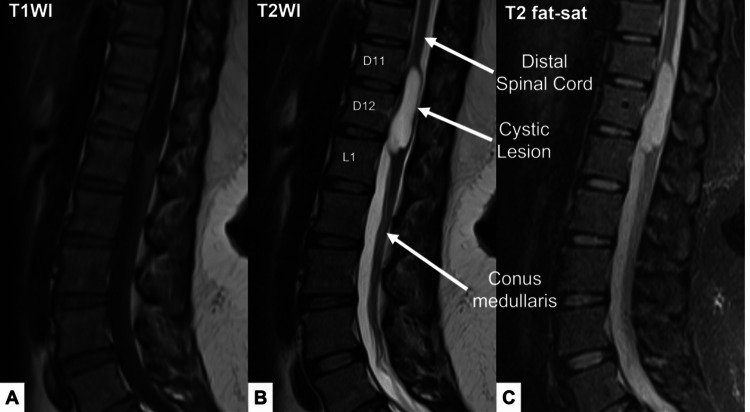
MRI of the patient - image 1 Sagittal and axial imaging with T1-weighted, T2-weighted, and T2 fat-saturated sequences reveal a lesion in the thoracic spine at the level opposite T11/T12 and the upper limit of the L1 vertebral body. The lesion is hypointense on T1 and hyperintense on T2 and T2 fat-saturated images, and it is causing expansion of the thoracic canal and displacement of the spinal cord MRI: magnetic resonance imaging

**Figure 2:**
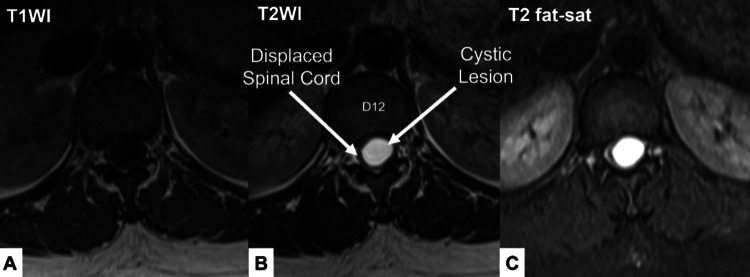
MRI of the patient - image 2 Axial images through the body of the T12 vertebra with T1-weighted (A), T2-weighted (B), and T2 fat-saturated (C) sequences showing a cystic lesion in the thoracic spine. The lesion is hypointense on T1 and hyperintense on T2 and T2 fat-saturated images MRI: magnetic resonance imaging

An MRI scan of the lumbosacral spine and dorsolumbar junction was performed. The MRI images revealed an intradural, extramedullary cystic lesion opposite T11/T12 and the upper limit of the L1 vertebral body in the thoracic spine. The lesion appeared hyperintense on T2 and hypointense on T1, and it was expanding the thoracic canal, pushing the cord to the side (Figures 1, 2). The findings were suggestive of post-spinal anesthesia intradural extramedullary arachnoid cyst formation extending between the T11 and L1 vertebral body, likely related to the spinal anesthesia given for the C-section six months ago.

## Discussion

Arachnoid cysts are benign cystic lesions that are usually asymptomatic and incidentally discovered in imaging studies. However, they can occasionally present with neurological symptoms such as back pain, leg numbness, and weakness, depending on their location and size. Intradural extramedullary arachnoid cysts can occur following spinal anesthesia, as a result of a dural puncture or inadvertent injection of local anesthetic or air into the subarachnoid space.

In our patient, the MRI images revealed an intradural, extramedullary cystic lesion opposite T11/T12 and the upper limit of the L1 vertebral body in the thoracic spine. A similar case has been reported by Samuel and Howell [2], which showed an epidural collection in the spinal canal extending from the lower end of the eighth thoracic vertebra to the upper end of the eleventh thoracic vertebra. In both of these cases, the patients were females of reproductive age who had received spinal anesthesia for a C-section.

The diagnosis of intradural extramedullary arachnoid cysts can be made through MRI imaging, which typically shows a well-defined cystic lesion with CSF signal intensity. The cyst is usually hyperintense on T2-weighted images and hypointense on T1-weighted images. The location of the cyst can also be identified on sagittal and axial images. In this case, the cyst was located between T11 and L1 vertebral body and was expanding the thoracic canal, pushing the cord to the side.

Treating primary and secondary extradural spinal cysts is generally easy and effective with cyst resection or fenestration. However, secondary intradural spinal arachnoid cysts (ISACs), which can occur after iatrogenic events, trauma, and subarachnoid hemorrhage, are difficult to treat because they are often large, ventrally located, and may contain arteriovenous malformations [12].

Despite the availability of various surgical techniques, there is currently no consensus on the optimal surgical management approach [6]. In most cases, complete surgical resection of the cyst, followed by closure of the communication pathway and dural defect repair, is a curative option that enhances neurological function [13,14]. Another treatment option is laminoplasty, which provides proper exposure and decompression of the spinal canal while maintaining spinal stability and the integrity of posterior elements [15]. Subarachnoid space cyst drainage is another treatment option, but it has been reported to yield only temporary results [8,16]. The surgical excision of the arachnoid cyst was carried out in our case, and the patient tolerated the procedure well, leading to substantial improvements in her symptoms.

## Conclusions

Post-spinal anesthesia intradural extramedullary arachnoid cyst formation is a rare but potentially serious complication of spinal anesthesia. MRI is the diagnostic tool of choice for the evaluation of arachnoid cysts, and surgical excision is the treatment of choice. The prognosis for patients with intradural extramedullary arachnoid cysts is generally good if the cyst is excised promptly. Physicians should be aware of this potential complication of spinal anesthesia and consider it in the differential diagnosis of patients with neurological symptoms following spinal anesthesia.

To minimize the risk of intradural extramedullary arachnoid cyst formation following spinal anesthesia, anesthesiologists should prioritize maintaining a sterile environment through strict aseptic techniques. Careful selection of the appropriate needle size and minimizing unnecessary manipulation of the needle within the subarachnoid space is crucial to reduce the likelihood of dural injury, which may contribute to the development of arachnoid cysts. By diligently implementing these preventive measures, anesthesiologists can significantly enhance patient safety during spinal anesthesia procedures and reduce the risk of this rare complication.
